# Paraganglioma of Urinary Bladder: An Uncommon Entity in Uropathology

**DOI:** 10.7759/cureus.17265

**Published:** 2021-08-17

**Authors:** Arpita Saha, Kaushik Saha, Nilanjan Sarkar, Ishfaq A Geelani

**Affiliations:** 1 Pathology, Manipal Tata Medical College, Manipal Academy of Higher Education, Jamshedpur, IND; 2 Pathology, Tata Main Hospital, Jamshedpur, IND; 3 Radiology, Tata Main Hospital, Jamshedpur, IND; 4 Urology, Royal Hospital, Muscat, OMN

**Keywords:** paraganglioma, catecholamines, urothelial carcinoma, immunohistochemistry, cystoscopy

## Abstract

Paraganglioma of the urinary bladder is a rare neoplasm. It may be functional, secreting catecholamines, or nonfunctional. Clinically and histopathologically, it has the potential to be misdiagnosed as a more common urothelial carcinoma, especially in nonfunctional cases. A high index of suspicion on the part of pathologist can help in identification of characteristic histopathologic feature which coupled with immunohistochemistry can help in establishing the correct diagnosis. We present a case of paraganglioma in a 78-year-old male patient presenting with haematuria. Clinical provisional diagnosis rendered based on cystoscopic findings and radiology was urothelial carcinoma; however, was confirmed to be a case of paraganglioma of bladder on histopathological and immunohistochemical evaluation. A long follow-up is warranted. Herein, we also briefly review the relevant literature.

## Introduction

Paraganglioma is an extra-adrenal pheochromocytoma. About 10% of paraganglioma occur in extra-adrenal sites, of which, 10% are located in bladder wall accounting for 0.05% of all bladder tumors. It arises from paraganglion cells of bladder wall [1]. Here, we report a case of large urinary bladder paraganglioma clinically suspected to be urothelial carcinoma.

## Case presentation

**Figure 1:**
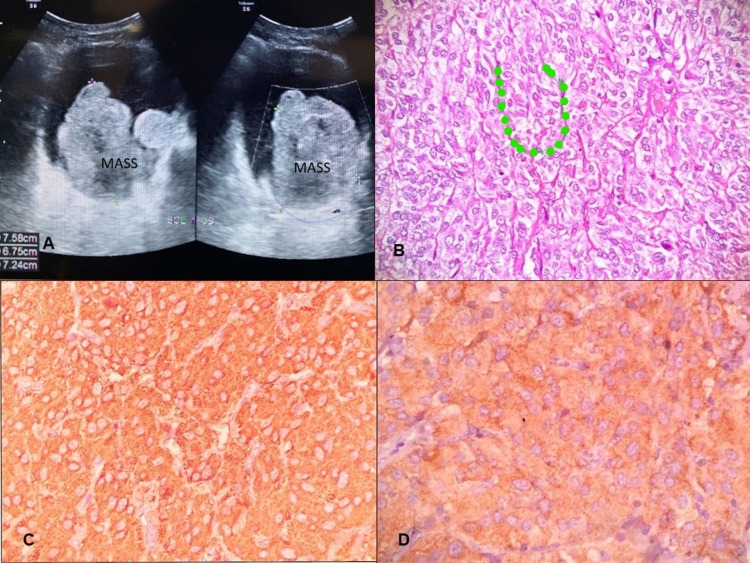
Radiomorphological and immunohistochemistry image of the bladder mass (A) Ultrasonography image showing a large polypoidal mass arising from the left lateral bladder wall. (B) Microphotograph showing nests of polygonal cells with thin capillary network investment at the periphery referred to as zellballen pattern; dotted line (hematoxylin and eosin 40x). (C) Immunohistochemistry for synaptophysin showing positive cytoplasmic staining in the tumor cells (DAB 20x). (D) Immunohistochemistry for chromogranin showing positive cytoplasmic staining in the tumor cells (DAB 40x). DAB: 3, 3'-diaminobenzidine

A 78-year-old male, farmer by occupation, presented to the urology outpatient department with a complaint of intermittent haematuria for seven days. There was no other significant history like fever, dysuria, abdominal pain, etc. Past medical/surgical history was insignificant except for inguinal hernia operation five years back. He was not known to be diabetic, hypertensive, hypothyroid, or hyperlipidemic. The patient had no history of headaches, palpitations, or dizziness associated with micturition or postural changes. There was no significant family history of similar disease. Laboratory results including routine microscopy and microbiological examination of urine were largely unremarkable except for the presence of mild hematuria (eight to 10 RBC/high power field). The rest of the hematological and biochemical tests were within normal range except for mild low hemoglobin (10.0 g). Physical examination was unremarkable and vitals were stable. Radiology (ultrasonography) image shows a large polypoidal lesion with inner echogenicity and smooth lobular surface. The lesion was large measuring 7.58 x 6.75 x 7.24 cm. Doppler scan shows the arterial supply of the lesion. A possibility of soft tissue/mesenchymal lesion was considered along with a possibility of urothelial carcinoma (Figure 1A). Cystoscopy revealed a large solid tumor arising from the left lateral wall of the urinary bladder. Transurethral resection of bladder tumor (TURBT) was done and the specimen was sent for histopathological examination. In the laboratory, the specimen was received in buffered formalin solution. The specimen was in multiple fragmented bits, gray tan in color. Microscopic evaluation showed a tumor disposed in nesting/zellballen pattern, formed by prominent intratumoral fibrovascular network, and sustentacular cells wrapping the nests of tumor cells. Individual cells were polygonal, with abundant granular eosinophilic to basophilic cytoplasm, uniform round to oval nuclei, regular nuclear outline, evenly dispersed granular chromatin, and inconspicuous nucleoli (Figure 1B). Focal clear cell changes were seen. Mitosis was inconspicuous. The tumor was seen to invade the muscularis propria/detrusor muscle. No high-grade areas or necrosis were seen. No chronic inflammatory infiltrate or epithelial atypia was seen. At places, nests of bland urothelium (von Brunn nest) and cystitis cystica were present. Immunohistochemistry showed tumor cells to be positive for synaptophysin, chromogranin, and sustentacular cells for S-100 (Figures 1C, 1D). Succinate dehydrogenase isoform B (SDHB) immunohistochemistry was suggested as a predictive marker, but the patient couldn’t afford it. The patient was in follow-up with regular cystoscopy, urinary, and blood catecholamine estimation (vanillylmandelic acid) for three years without any recurrence. Urinary catecholamine estimation (vanillylmandelic acid {VMA} and homovanillic acid {HVA}) was not done, as the possibility of paraganglioma was not considered on a prior basis. The postoperative VMA level was 8.3 mg/24 h of urine.

## Discussion

Pheochromocytomas are neuro-endocrine neoplasms originating from tissue of the autonomic nervous system, mostly located in the adrenal cortex. Paragangliomas (PG) are extra-adrenal pheochromocytomas arising from embryonic nests of chromaffin cells from the sympathetic plexus. In the urinary bladder, they arise from the chromaffin cells located in detrusor muscle of the bladder wall [2]. Ten percent of the PGs occur in the extra-adrenal site and the urinary bladder is a rare site of PG accounting for 10% of extra-adrenal PGs. Also, PG in the bladder is extremely rare bladder tumor accounting for less than 0.05% of all bladder tumors and was first described by Zimmerman in 1953 [1,3].

Being a rare entity, it has propensity to be misdiagnosed as urothelial cancer; however, characteristic histomorphological and immunohistochemical features aid in the correct diagnosis. The reasons behind its misdiagnosis being (1) frequent involvement of the muscularis propria (detrusor muscle) layer; (2) morphology mimics urothelial carcinoma particularly in transurethral resection specimens, especially, if there are artifactual changes induced by surgical cautery; (3) failure to include it in histological differential diagnosis while evaluating a bladder tumor; and (4) only a minority of patient present with catecholamine-associated symptoms that might prompt consideration of the diagnosis [4].

It is very critical to distinguish paraganglioma from urothelial carcinoma because of potential differences in therapy as well as prognosis. Treatment modalities for urothelial carcinoma are dependent on the stage of the disease. For non-muscle invasive carcinomas intravesical bacillus Calmette-Guerin (BCG), surveillance and reTURBT are commonly done whereas for muscle-invasive urothelial carcinoma requires aggressive treatment in form of radical cystectomy. On the other hand, TURBT/partial cystectomy with complete removal of tumor is treatment of choice in PG, even if the muscles are invasive. Chemotherapy and radiotherapy may be required in rare metastatic settings [5,6].

Clinical features suggesting the occurrence of PG are related to catecholamine secretion such as episodic or sustained hypertension, hypertensive crisis during micturition, headache, blurred vision, and also hematuria. Eighty-three percent of the paragangliomas in the bladder are hormonally active [2]. However, characteristic clinical features are present in minority of cases. Zhou et al. reported only 13% cases of their series with catecholamine-related symptoms, while Menon et al. reported the incidence is 7% (one out of 14 cases). Intraoperative hypertensive crisis reported by Menon et al. may be catastrophic, hence preoperative high index of suspicion, especially, in cases with atypical cystoscopic finding is required [6]. Preoperative catecholamine level estimation may be helpful in such situations.

Our case presented with hematuria and no feature of catecholamine release was documented. Also, follow-up in our case by radiology (CT) was done, which showed no tumor mass elsewhere in the body.

Age distribution of PG is one to two decades younger than the average age of urothelial carcinoma, median age being 43 years and 45 years. It can affect any part of the urinary bladder wall with predilection to the dome and trigone of the bladder, and in almost half the cases, muscularis propria is involved [1,7]. However, the index case was in an elderly male.

Histologically characteristic zellballen pattern with nest delineated by thin fibrovascular septae should be present at least focally for diagnosis of PG. If not found in a first go, a diligent search is advocated. Tumor cells in PG are large epithelioid with abundant eosinophilic/amphophilic and granular cytoplasm, regular monomorphic nuclei. A rare bizarre nuclear atypia is acceptable however. Muscle involvement in PG is characterized by entrapped tumor cells within the muscle bundles without desmoplasia, the presence of later favor invasive urothelial carcinoma [4]. Rare mitosis, necrosis, and vascular invasion can be seen [1].

The main differential considered is urothelial carcinoma, a nested variant. The nested variant of urothelial carcinoma is characterized by confluent small nests and abortive tubular growth pattern. Individual tumor cells are mildly atypical with irregular infiltrating tumor-stroma interface. This variant is prognostically aggressive. Immunohistochemistry is useful in differentiating, urothelial carcinoma express cytokeratin and other epithelial markers while PG express neuroendocrine markers, eg., synaptophysin, chromogranin, S100P (sustentacular cells) [8-10]. Other differentials considered are metastatic renal cell carcinoma, prostatic carcinoma involving bladder, metastatic melanoma, carcinoid, and granular cell tumor [4,11]. A detailed discussion of differentials is out of scope of this report. However, characteristic histomorphology coupled with immunohistochemical profile helps in establishing the diagnosis. Also, in suspected case, keeping the specimen in Zenker's fluid may show chromaffin reaction, which gives clue for the diagnosis. Our case was not kept in Zenker's solution.

Genetically, PG are heterogeneous with frequent loss of 1p, 3q, and 22q [1]. Thirty percent of paragangliomas are estimated to be familiar with germline mutations associated with von-Hippel Lindau (VHL) syndrome, multiple endocrine neoplasia, type 2 (MEN2) syndrome, neurofibromatosis 1 (NF1) syndrome, and familial paraganglioma-pheochromocytoma syndromes (SDH gene). Genetic study for these germline mutations along with IHC for SDHB should ideally be undertaken in these tumors. Extra-adrenal paragangliomas with SDH mutation are more likely to have malignant potential [6]. No genetic syndromic study was done in our case; however, the patient did not have any family history of paraganglioma syndrome complex.

## Conclusions

To conclude, urinary bladder paraganglioma is a rare entity. It is mostly unsuspected preoperatively. A proportion of the cases may show hypertensive crisis/syndromic association and gives clinical clue to the diagnosis, however, a significant majority of cases are detected incidentally after cystoscopic biopsy. Hence, both pathologist and clinician should have a high index of suspicion to have a proper diagnosis and treatment. A proper family screening and search for other neoplastic association to be followed.
